# Spherical-supported membranes as platforms for screening against membrane protein targets

**DOI:** 10.1016/j.ab.2018.03.006

**Published:** 2018-05-15

**Authors:** V. Vasilca, A. Sadeghpour, S. Rawson, L.E. Hawke, S.A. Baldwin, T. Wilkinson, D. Bannister, V.L.G. Postis, M. Rappolt, S.P. Muench, L.J.C. Jeuken

**Affiliations:** a The School of Biomedical Sciences and the Astbury Centre for Structural Molecular Biology, University of Leeds, Leeds, LS2 9JT, United Kingdom; b School of Food Science and Nutrition, University of Leeds, Leeds, LS2 9JT, United Kingdom; c Center for X-ray Analytics, Department of Materials Meet Life, EMPA, 9014 St Gallen, Switzerland; d MedImmune, Antibody Discovery and Protein Engineering, Milstein Building, Granta Park, Cambridge, CB21 6GH, United Kingdom; e Biomedicine Research Group, Faculty of Health and Social Sciences, Leeds Beckett University, Leeds, United Kingdom

## Abstract

Screening assays performed against membrane protein targets (e.g. phage display) are hampered by issues arising from protein expression and purification, protein stability in detergent solutions and epitope concealment by detergent micelles. Here, we have studied a fast and simple method to improve screening against membrane proteins: spherical-supported bilayer lipid membranes (“SSBLM”). SSBLMs can be quickly isolated via low-speed centrifugation and redispersed in liquid solutions while presenting the target protein in a native-like lipid environment. To provide proof-of-concept, SSBLMs embedding the polytopic bacterial nucleoside transporter NupC were assembled on 100- and 200 nm silica particles. To test specific binding of antibodies, NupC was tagged with a poly-histidine epitope in one of its central loops between two transmembrane helices. Fluorescent labelling, small angle X-ray scattering (SAXS) and cryo-electron microscopy (cryo-EM) were used to monitor formation of the SSBLMs. Specific binding of an anti-his antibody and a gold-nitrilotriacetic acid (NTA) conjugate probe was confirmed with ELISAs and cryo-EM. SSBLMs for screening could be made with purified and lipid reconstituted NupC, as well as crude bacterial membrane extracts. We conclude that SSBLMs are a promising new means of presenting membrane protein targets for (biomimetic) antibody screening in a native-like lipid environment.

## Introduction

Encoded by almost one third of archaean, bacterial and eukaryote DNA [1], membrane proteins represent vital cellular components for all lifeforms. Given their essential roles towards sustaining life, it is unsurprising that membrane protein pathology accounts for a large number of debilitating conditions, such as Bartter syndrome, cardiac arrhythmia and hypertension, congenital deafness and myotonia, cystic fibrosis, epilepsy, osteoporosis and polycystic kidney disease [2,3]. Their significant therapeutic importance has led to many of today's pharmaceuticals targeting membrane proteins [4,5], with the largest class being the G-protein coupled receptors (GPCRs). However, the discovery of novel membrane protein binders – including antibody-based medicines that have emerged throughout the last decade [6] – is not without issue. The high-throughput protocols employed by the drug discovery industry demand high levels of expression and purity from their designated screening targets, yet few membrane proteins can be expressed at high level within their native membranes. Moreover, the general study of membrane proteins is further complicated by the fact that advanced research techniques (e.g., kinetic and ligand-binding characterisation, nuclear magnetic resonance (NMR) or X-ray crystallography) cannot always be directly performed on crude cellular membranes and thus require generous amounts of recombinant protein of high purity and conformational stability, therefore becoming reliant on identifying optimised expression platforms, a suitable detergent for the solubilisation and, more often than not, demanding high-throughput methodologies [[7], [8], [9]].

Unfortunately, systems used in the overproduction of membrane protein targets rarely express high amounts of recombinant protein [10], partly due to differences between the biogenesis pathways of the host and those of the expression systems and/or the imposed xenobiotic toxicity [8]. Even following successful expression, membrane proteins are notoriously difficult to purify via standard techniques such as ion exchange or hydrophobic interaction and poor overall yields can still be registered after the inclusion of specialised high-affinity chromatography tags [11]. Furthermore, target denaturation is an ever-present concern after the proteins have been removed from their native membranes and this is the main reason why detergent solubilisation has been traditionally used to counter the considerable hydrophobicity and aggregation tendency of membrane proteins post-purification [7,9]. While detergent-solubilised proteins facilitate screening with other biomolecules such as ligands or inhibitors in solution [12], it is commonly desirable to transfer the target proteins into less disruptive environments, since even the mildest detergents can still lead to the complete inactivation of the solubilised proteins [7]. Moreover, in the context of antibody binding studies, detergent micelles can also actively block potential epitopes on the chosen screening targets and can thus have a direct negative impact on the discovery of new antibody-based pharmaceuticals [12,13].

The main objective of the research presented here was therefore to develop an alternative screening platform based on spherical-supported bilayer lipid membranes (“SSBLMs”), which can present membrane protein targets in a native-like lipid environment. SSBLM consist of a solid spherical core, typically silica, which is coated with lipid membranes. SSBLMs were first developed in the 80s and 90s, are well characterised with spectroscopy and microscopy and their formation has been well documented (see Ref. [14] for a review on SSBLMs). SSBLMs have already been reported for several membrane proteins, such as the multidrug efflux pump component OprM [15], bacteriorhodopsin [16] or the redox-driven proton pump cytochrome *c* oxidase [17]. This prompted us to explore whether, by refinement of the SSBLM format, this technology can be used in assays that require or select for specific, high-affinity antibody binding and, eventually, screening assays. In order to enhance the amount of protein presented in a screening assay, submicron silica particles were used.

In order to provide proof-of-concept for our proposed screening platform, the bacterial nucleoside transporter NupC was chosen as the membrane protein of interest. Involved in active (secondary) transport of both purine and pyrimidine nucleosides across bacterial inner membranes (IMs), NupC is a proton-dependent symporter belonging to the concentrative nucleoside transporter (CNT) family [[18], [19], [20]]. The protein shares 22–26% amino acid sequence identity with the human transporters hCNT1-3, which renders it a good model for studying the transport of the therapeutic nucleoside analogues used in the treatment of life-threatening viral and neoplastic diseases, such as azidothymidine and gemcitabine [21]. Since antibody-based pharmaceuticals are typically expected to target epitopes located in the loop regions of transmembrane proteins, a clone of NupC was engineered to feature a His-tag on one of its central loops, between two transmembrane helices. This affinity tag allowed for the binding of both anti-His antibodies as well as gold-conjugated nitrilotriacetic acid (NTA) probes, which greatly aided us in providing our proof-of-concept.

Here, we show that SSBLMs are a suitable platform for screening assays and report on technical improvements that are required to reduce non-specific binding of antibodies to the SSBLM particles. Non-specific binding of proteins, including antibodies and biomimetic antibodies, can occur if silica particles are not completely coated by the lipid membranes, exposing some of the bare silica surface [22]. Here, we show that including liposomes and bovine serum albumin (BSA), but not detergents, during the incubation steps with antibodies is a simple and effective strategy to block non-specific binding. Furthermore, we show that this method can also be applied when using crude membrane extracts, negating the need to tag and purify membrane proteins in screening assays.

## Materials and methods

### Materials

All chemicals were purchased from Sigma-Aldrich or Melford unless stated otherwise. His-tagged NupC detection employed HRP-conjugated mouse IgG_1_ anti-His antibodies (R&D Systems, MAB050H). Lipid, detergent and related materials included 1-palmitoyl-2-oleoyl-*sn*-glycero-3-phosphocholine (POPC) lipids (Avanti Polar Lipids, 850457), *α*-[4-(1,1,3,3-Tetramethylbutyl)phenyl]-*w*-hydroxy-poly(oxy-1,2-ethanediyl) (Triton X-100) (10% (w/v) solution) (Anatrace, APX100), Bio-Beads SM-2 adsorbent beads (Bio-Rad, 1523920) and Texas Red 1,2-Dihexadecanoyl-*sn*-glycero-3-phosphoethanolamine triethylammonium salt (TR-DHPE) (Thermo Fisher Scientific, T1395MP). Silicon dioxide (SiO_2_) spheres with diameters of 100- and 200 nm were supplied as 10 mg/mL aqueous solutions (nanoComposix, SISN100 and SISN200, respectively). The peroxidase assay employed a SensoLyte 10-Acetyl-3,7-dihydroxyphenoxazine (ADHP) peroxidase assay kit (fluorimetric) (AnaSpec, AS-71111). Cryo-EM materials included 5 nm Ni-NTA-Nanogold probes (Nanoprobes, 2082) and lacey carbon film/copper mesh cryo-grids (Agar Scientific, AGS166).

### NupC cloning

Both an untagged version (pGJL16) as well as a His-tagged construct, of NupC (pLH13), were used. The plasmid pGJL16 was obtained by cloning the *E. coli nupC* gene into a pTTQ18 vector [23] between *Eco*RI and *Hin*dIII. pTTQ18 features an isopropyl β-D-thiogalactopyranoside (IPTG)-inducible *tac* promotor [23]. pLH13 was then cloned from pGJL16 through the insertion of a pentahistidine tag. We previously reported that cloning a His-tag into either the N- or C-terminus of NupC prevents its expression [24], hence a pentahistine tag was inserted in the central cytoplasmic loop between transmembrane (TM) helices 5 and 6, specifically between His230 and Glu231. The pentahistine tag, along with the native His230, thus resulted in 6 consecutive histidines. In pLH13, Cys96 was also mutated to an Ala to reduce potential dimerisation and aggregation. While the uridine uptake activity of the internally His-tagged NupC construct was substantially reduced compared to the wild-type variant, its post-purification functionality was nevertheless retained (unpublished results).

### Purification of His-tagged NupC

The purification of the His-tagged NupC was modified from Ref. [24]. pLH13 was transformed into *E. coli* strain C43 and grown in Lysogeny broth (LB) media supplemented with 100 μg/mL carbenicillin. C43/pLH13 was cultured as 500 mL cultures in 2 L baffled flasks at 37 °C with 200 rpm orbital shaking until reaching an OD_600nm_ of ∼0.6, after which expression was induced with 1 mM IPTG (Generon) for another 4 h. The cells were then harvested via centrifugation (9000×*g* for 20 min) and resuspended in 20 mM Tris, 0.5 mM EDTA (pH 7.4) using volumes five to six times the weight of the harvested cells. Once resuspended, the cells were homogenised using an Ultra-Turrax cell homogeniser and subsequently lysed via two consecutive runs through a TS5/40/AB/GA cell disrupter (Constant Systems) at 30 kPsi. The lysed cells were centrifuged at 14,000×*g* for 45 min in order to remove cellular debris. The supernatant was ultracentrifuged at 131,000×*g* for 2 h to isolate the bacterial membranes. The protein concentration of the membrane preparation was determined using the bicinchoninic acid (BCA) assay. The membranes were solubilised in solubilisation buffer (1% (w/v) n-Dodecyl-β-D-maltoside (DDM), 50 mM phosphate buffer, 10% (w/v) glycerol, 150 mM NaCl, 5 mM imidazole and cOmplete™ (EDTA-free) mini protease inhibitor cocktail, pH 7.4) at 4 °C for 1 h at a total membrane protein concentration of approximately 5 mg/mL. The solubilised membranes were then ultracentrifuged at 110,000×*g* for 1 h, after which the insoluble pellet was discarded. The supernatant was added to a bed volume of 80 μL of cobalt affinity chromatography resin (Pierce) per 25 mg of total membrane protein, pre-equilibrated in wash buffer (50 mM phosphate buffer, 10% (w/v) glycerol, 150 mM NaCl, 5 mM imidazole and 0.05% (w/v) DDM, pH 7.4). NupC was bound to the resin for 16 h at 4 °C with gentle roller mixing. The resin was packed into a disposable filtered column (Thermo-Pierce Scientific) and washed at 20 °C with 10 column volumes of wash buffer. NupC was eluted in 0.5 mL fractions using elution buffer (50 mM phosphate buffer, 10% (w/v) glycerol, 150 mM NaCl, 300 mM imidazole and 0.05% (w/v) DDM, pH 7.4) and subsequently dialysed for another 16 h at 4 °C against dialysis buffer (50 mM MES, 10% (w/v) glycerol, 150 mM NaCl and 0.05% (w/v) DDM, pH 6.8). Finally, the protein samples were concentrated using a Vivaspin concentrator (Sartorius) with a 10 kDa molecular weight cut off (MWCO). The concentrated NupC was snap-frozen in liquid nitrogen and stored at −80 °C.

### Liposome preparations

POPC was dissolved in chloroform, distributed into 5 mg aliquots and dried first under a nitrogen stream, then under vacuum for 2 h. The desiccated lipid aliquots were stored under a nitrogen atmosphere at −20 °C until used. Liposomes were prepared by first rehydrating the above-mentioned aliquots in phosphate-buffered saline (PBS), typically at concentrations of 5 mg/mL. The lipid suspensions were then passed 11 times through a fully assembled Mini-Extruder (Avanti Polar Lipids), fitted with a polycarbonate track-etched membrane (Whatman) featuring either 100 nm or 200 nm pore sizes, sandwiched between four extruder drain discs (i.e. two on each side of the membrane). The fluorescent labelling of POPC liposomes was achieved by first dissolving Texas Red (TR)-modified lipids in a 1:1 (v/v) mixture of chloroform and methanol (0.5 mg/mL) and adding 100 μL of it to a 5 mg POPC aliquot (i.e. 1% (w/w)) prior to performing the drying and extrusion steps described above.

### Inner membrane extract preparation

C43/pGJL16 was cultured as described above and the total membrane fraction was isolated as for C43/pLH13. Following ultracentrifugation, the membrane pellet (i.e., the total membrane extract) was resuspended in a 25% (w/w) sucrose Tris/EDTA buffer (20 mM Tris/HCl, 0.5 mM EDTA, pH 7.5). A 30–55% (w/w) sucrose gradient with centrifugation at 131,000×*g* for 16 h was used to separate the inner membrane (IM) from the outer membrane [25]. The IM fraction was collected from the gradient and washed with Tris/EDTA buffer via two other 1-h centrifugations at 131,000×*g*. The protein concentration of the IM fraction was determined via BCA assay, after which the IM vesicles were resuspended in Tris/EDTA buffer and stored in 5 mg/mL protein aliquots at −80 °C. For SSBLM formation, the IM vesicles were mixed with POPC liposomes at various ratios expressed as protein weight of the IM versus dry lipid weight of the POPC vesicles. The resulting IM/POPC mixture was then snap-frozen in liquid nitrogen and thawed by immersing the test tube in water at 20 °C. This freeze-thaw procedure was repeated three times after which the IM/POPC mixture was extruded through a 200 nm track-etched membrane as described above.

### NupC reconstitution

Reconstitution of His-tagged NupC into proteoliposomes was performed following a modified method of Geertsma et al. [26]. POPC liposomes (5 mg/mL) in PBS were prepared as detailed above using 200 nm track-etched membranes. 1 mL of liposomes was titrated with 10% (w/v) Triton X-100 until R_sat_ was reached (as monitored by an increase in OD_540 nm_), after which an additional 5 μL of Triton X-100 were added. NupC was mixed in at a protein-lipid ratio of between 1 and 2.4% (w/w) and incubated for 15 min at 20 °C. Bio-Beads SM-2 (50 mg) were added to remove Triton X-100 from solution during a 30 min incubation at 20 °C under gentle roller mixing. This step was repeated twice using 60 min and 16 h incubations at 4 °C, after which the proteoliposomes were harvested via ultracentrifugation (100,000×*g* for 1 h at 4 °C) and resuspended in PBS. Finally, the proteoliposomes were re-extruded through 200 nm track-etched membrane as described above.

### SSBLM formation

Silica particles (typically 250 μg) were vigorously vortexed with liposomes at different lipid-to-particle ratios (w/w), as indicated in the Results section. Following a 1 h incubation at 20 °C with gentle roller mixing, the resulting SSBLMs were pelleted via centrifugation (1 min 17,000×*g*). The supernatants were removed (or transferred into separate tubes, if required), while the particle pellets were washed by vortexing in identical volumes of deionised water, followed by a 30 min incubation at 4 °C with gentle roller mixing to remove any unbound lipid materials. The washed SSBLMs were once again harvested by centrifugation and resuspended in PBS prior to being used or stored at 4 °C.

Standard procedures were used for SDS-PAGE [27] and Western blot analysis [28]. For Western blot analysis, SSBLM samples were mixed with SDS-PAGE loading buffer (containing SDS) and incubated for 1–2 h at 37^°^ degrees to solubilise the membranes and embedded proteins. The silica particles were removed by a short spin (1 min 17,000×*g*) and the SDS-PAGE loading buffer (supernatant) was used to load on the SDS-PAGE.

### Cryo-electron microscopy (cryo-EM)

100 μL SSBLM samples were created as described above by mixing His-tagged NupC/POPC proteoliposomes (2% (w/w) protein/lipid ratio) with 200 nm silica particles at a 25% (w/w) liposome/particle ratio. Protein-free particles were also formed at equivalent concentrations to serve as negative controls. Following the deionised water wash, the SSBLM samples were pelleted and resuspended in 100 μL volumes of Ni-NTA-Nanogold probe solution, prepared to a 10:1 probe/protein molar ratio in blocking buffer, consisting of PBS supplemented with 50 μg/mL POPC vesicles and 1 mg/mL bovine serum albumin (BSA). After a 30 min incubation at 4 °C with gentle roller mixing, the SSBLMs were pelleted and washed twice via vortexing, first in blocking buffer, then in regular PBS, before being diluted 10× further with PBS and applied to the cryo-EM grids. These were prepared using a FEI Vitrobot Mark IV by first applying 3 μL of sample per grid (which had previously been glow-discharged for 40 s), then blotting off the excess solution for 2 s and finally plunge-freezing the grids in liquid ethane. All of the prepared cryo-grids were stored in liquid nitrogen prior to being imaged. The grids were imaged at a magnification of 35,000× using a FEI Tecnai F20 transmission electron microscope (TEM) fitted with a Gatan 4 K × 4 K charge-coupled device (CCD) camera. All of the images were collected in “low-dose” mode.

### Small angle X-ray scattering (SAXS)

All small angle X-ray scattering (SAXS) measurements were performed at 20 °C. The operated SAXS camera setup (SAXSpace, Anton Paar, Austria) is described in great detail elsewhere [29]. Briefly, a high-resolution mode was chosen that allowed for the detection of a minimum scattering vector, q_min_, of 0.04 nm^−1^ (q = (4π/λ) sinθ, where 2θ is the scattering angle and λ is the wavelength of the X-ray beam, namely 0.154 nm). All studied samples were filled into the same vacuum-tight, reusable 1 mm quartz capillary, in order to give the exact same scattering volume each time. A Mythen X-ray detector system (Dectris Ltd., Baden, Switzerland) was used for recording the 1D scattering patterns. SAXStreat software (Anton Paar, Graz, Austria) was used to refine the primary beam position. Background scattering from water and capillary was subtracted using the SAXSQuant software (Anton Paar, Graz, Austria).

Background subtracted SAXS patterns were analysed using an extended core shell model [30]. This model provides the scattering from a spherical core (silica) and six concentric shell structures: five used to build up the POPC bilayer and one for the space between the silica sphere and the lipid bilayer (i.e. the intermediate water layer). All electron densities were fixed to literature values [31,32], with ρ(silica) = 0.70 e/Å^3^, ρ(water) = 0.33 e/Å^3^, ρ(head group) = 0.45 e/Å^3^, ρ(CH_2_) = 0.30 e/Å^3^ and ρ(methyl) = 0.16 e/Å^3^. Additionally, the methyl trough and head group thicknesses were fixed to 0.5 nm and 0.8 nm, respectively. Thus, only three fitting parameters were considered: (i) the silica radius, (ii) the intermediate water thickness and (iii) the hydrocarbon chain length. The porosity of the Si-particles was taken into account by determining the form factor contribution from the nano-pores. For SAXS measurements, both SSBLM and negative control (i.e. bare silica particle) solutions were created at concentrations of 30 mg/mL in deionised water. In order to get statistically reliable data and increased signal to noise ratio, we acquired 12 scattering frames each with 30 min exposure time and computed the average curve for the further analysis.

### Enzyme-linked immunosorbent assay (ELISA)

Freshly-made SSBLM particles, resuspended in either regular PBS buffer or blocking buffer (i.e. PBS supplemented with 50 μg/mL POPC vesicles and 1 mg/mL BSA), were transferred to V-bottomed 96-well plates and subsequently incubated with 100 μL volumes of HRP-conjugated anti-His antibodies (diluted 1:5000 (v/v) in PBS buffer) for 1 h at 20 °C either in PBS or blocking buffer (10 μg/mL POPC vesicles and 1 mg/mL bovine serum albumin (BSA)) in conical 96-well plates. The particles were pelleted via centrifugation (3000×*g*, 2 min) and washed twice in 100 μL volumes of PBS buffer (first with and then without 50 μg/mL POPC liposomes (10 min incubations at 20 °C). The washed SSBLM pellets were resuspended in 50 μL of PBS buffer and transferred onto a flat-bottomed 96-well plate. Finally, each test well was supplemented with 50 μL of peroxidase assay working reagent (10-Acetyl-3,7-dihydroxyphenoxazine, ADHP) and incubated for 30 min at 20 °C before the reaction was stopped through the addition of equivalent volumes of 0.5 M H_2_SO_4_. Fluorescence was measured at 590/10 nm in a fluorescence plate reader, with excitation set to 545/10 nm.

## Results

### SSBLM formation

**Fig. 1:**
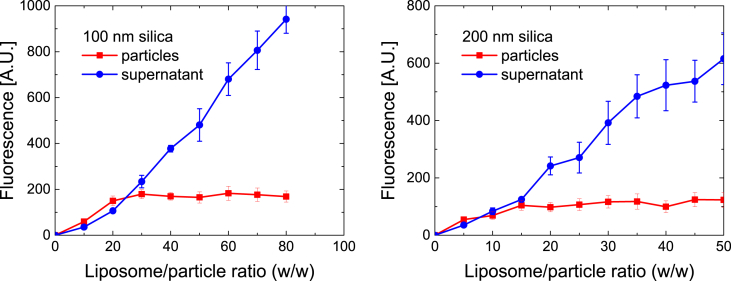
The fluorescence emissions (A.U.) resulting from 100 nm (left) and 200 nm (right) silica particles enveloped in fluorescently-labelled POPC SSBLMs (red), as well as from the supernatants obtained after pelleting the unwashed particles (blue). The vesicle/particle ratio is given in weight percent. The error bars represent the standard error of the mean, n = 2. (For interpretation of the references to colour in this figure legend, the reader is referred to the Web version of this article.)

100- and 200 nm silica particles were mixed with fluorescently-labelled POPC vesicles (100 nm) at different ratios to determine the saturation thresholds resulting in full lipid bilayer coverage of the particles (Fig. 1).

These measurements revealed that a minimal vesicle/particle ratio of 30% (w/w) was necessary to saturate the 100 nm silica particles, whereas their 200 nm counterparts appeared saturated beyond a ratio of 15% (w/w). Such behaviour is expected considering that the surface-to-volume ratio of particles scales linearly with their radius. Thus, 200 nm particles will have half the surface area of 100 nm particles when normalised to the weight of silica. Consequently, about half the amount of lipid material is needed to create SSBLMs on 200 nm particles compared to 100 nm particles.

While spectrofluorometry proved useful towards indicating whether the POPC lipids were adhering to the silica particles to the point of saturation, the results could not discriminate between correct SSBLM formation and the simple attachment of lipid material to the available silica surface. Therefore, small angle X-ray scattering (SAXS) and cryo-electron microscopy (cryo-EM) were used for a more detailed characterisation of the SSBLM particles. EM experiments are described below for NupC-embedding SSBLMs.

**Fig. 2:**
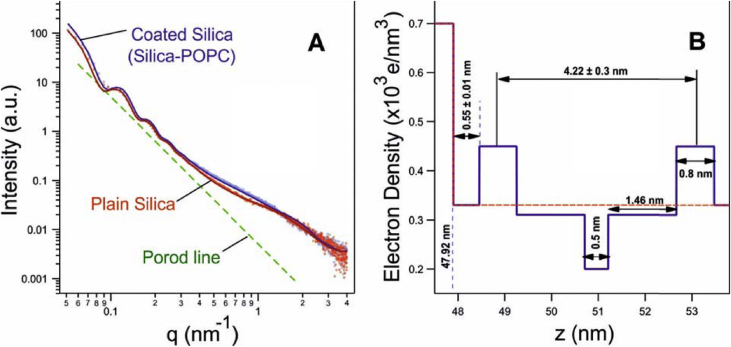
SAXS analysis of bare and POPC-coated silica particles. (A) The scattering profiles resulting from stock R ∼ 48 nm silica particles (red) and POPC SSBLMs (purple) alongside their corresponding fit functions (solid lines). The green dashed line shows the linear decay based on Porod's law for scattering from ideal spheres. (B) Refined electron density profile of our applied SSBLM model. (For interpretation of the references to colour in this figure legend, the reader is referred to the Web version of this article.)

The global fitting analysis of the SAXS data comparing the bare silica to the POPC-coated particles confirmed a proper and intact lipid bilayer coating. First, the bare silica particles radius (R) was determined with a value of 47.9 ± 3.5 nm, consistent with the manufacturer's specifications (Fig. 2A, red). Remarkably, a detailed look into the SAXS profiles of the bare silica particles reveals further a deviation from the expected Porod scattering of spheres (Fig. 2A, green). Note, the observed additional weak and broad scattering around *q* = 1.7 nm^−1^ is the form factor contribution arising from nano-pores (R_g_ = 1.1 nm) within the Si-particles.

Secondly, the SAXS data from the POPC-coated samples were then fitted with a fixed silica particle radius of 47.9 nm applying an extended core shell model (see Materials and Methods; Fig. 2A, purple). Not to over-parametrize the model, we kept the number of fitting parameters as low as possible. Hence, all commonly known electron densities of the modelled SSBLM layers were set to literature values and further the methyl trough and head group thicknesses were also fixed to 0.5 nm and 0.8 nm, respectively [31,32]. This means, only the radial dimensions of the intermediate water layer thickness and the hydrocarbon chain length were kept as free fitting parameters (Fig. 2B). The fitting results are shown in panel A as solid lines and are in excellent agreement with the recorded data points. The scattering contribution of the POPC bilayer coating is most dominant in the range of 0.4 < *q* < 1.1 nm^−1^, in which its form factor scattering contribution is recorded. Note, this *q*-range is well separated from the highest scattering contribution arising from the silica nano-pores, and hence, the evaluation of bilayer structure is unproblematic. In conclusion, the SAXS data analysis supports the proper and intact formation of the SSBLMs displaying a bilayer phosphate-to-phosphate distance of the supported lipid bilayers to be 4.2 ± 0.3 nm at 20 °C, which within errors agrees with the previously reported value of 3.9 nm by Kučerka et al. [33]. The fit also included the water layer for which a thickness of 0.55 ± 0.01 nm was determined, thinner than the 1.7 nm determined by Bayerl et al. by NMR for SSBLMs [34]. However, others have reported a large spread in the thickness of the water layer on planer substrates (e.g., compare ref [35] with [36]), some with a thickness between 0.2 and 0.8 nm [36].

### Protein incorporation into SSBLMs

**Fig. 3:**
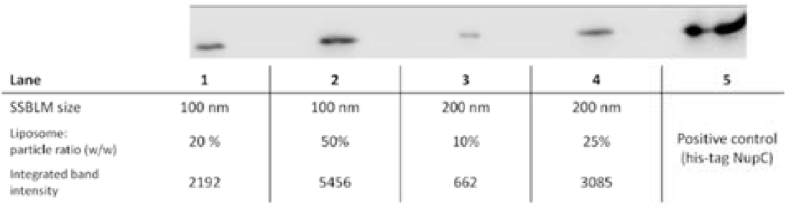
Western blot of 100- and 200 nm POPC SSBLMs embedding His-tagged NupC. SSBLMs were prepared as described in the text at different liposome/silica particle ratios as indicated in the Figure. Identical weights of silica are used in the Western blot. Purified His-tagged NupC was used as positive control in lane 5 and quantitative band intensities are given.

In order to embed membrane proteins into SSBLMs, fresh samples were formed using NupC-containing proteoliposomes. His-tagged NupC was first reconstituted into POPC vesicles at a 2.4% (w/w) protein/lipid ratio and the resulting proteoliposomes were subsequently used in the formation of both 100- and 200 nm NupC-embedding SSBLMs, as described under Materials and Methods. The successful embedding of NupC into the SSBLM was initially confirmed via SDS-PAGE and western blotting, using an HRP-conjugated anti-His antibody (Fig. 3). The appearance of the bands confirmed that NupC was indeed present within the SSBLMs, while the intensity of the NupC bands from the 100 nm SSBLM samples was significantly more intensive than that of the 200 nm SSBLM, as expected, given the proportionate increase in surface area (both SSBLM sample sizes used an identical amount of silica particles, so the 100 nm particles have double the surface area of the 200 nm).

### Specific binding of antibodies and gold-conjugated Ni-NTA probes

**Fig. 4:**
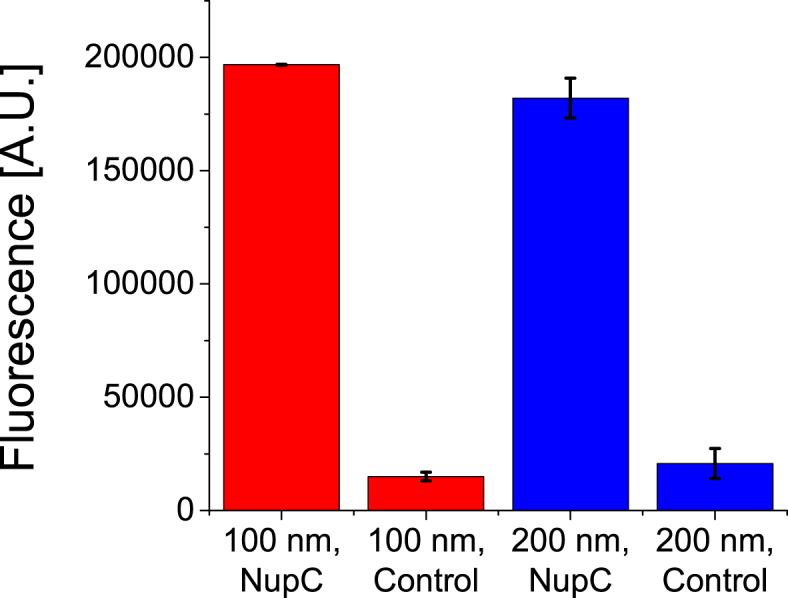
ELISA results (fluorescence signals emitted) from 100 nm (red) and 200 nm (blue) silica particles enveloped in protein-free POPC SSBLMs (control) or SSBLMs embedding His-tagged NupC (NupC). Control and NupC SSBLMs were treated identically. The error bars represent the standard error of the mean, n = 2. (For interpretation of the references to colour in this figure legend, the reader is referred to the Web version of this article.)

The suitability of SSBLMs for selective screening was tested through a peroxidase ELISA assay. Protein-free SSBLM particles were created for negative control purposes. It should be noted that, in order to preserve the structure of the SSBLMs, the antibody incubation step was performed in the absence of detergents typical of traditional ELISAs (i.e. Tween-20). Initial results revealed high background signals, due to non-specific binding of the antibody to SSBLM, presumable as a consequence of defects in the membrane coating, exposing the bare silica [22]. However, non-specific binding of antibodies could be blocked via the addition of 50 μg/mL POPC liposomes and 1 mg/mL BSA during the antibody incubation step. The 50 μg/mL POPC vesicles were added to all of the subsequent washing steps to repair any defects in the membrane layer that might arise during the assay [22]. The results obtained using this optimised protocol minimised non-specific antibody binding and confirmed that SSBLM can be used to bind (and hence screen) antibodies to membrane proteins in native-like lipid environment (Fig. 4).

**Fig. 5:**
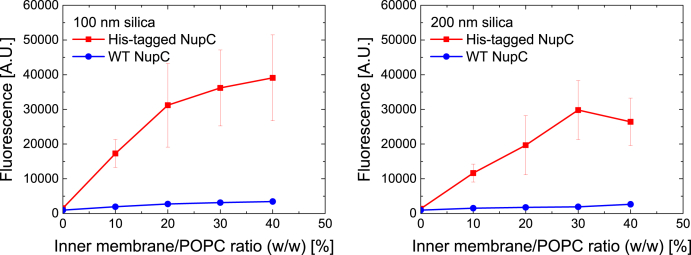
ELISA results (fluorescence signals emitted) from 100 nm (left) and 200 nm (right) silica particles enveloped in untagged NupC-expressing total membrane extracts (blue), as well as IM extracts overexpressing His-tagged NupC (red). Both IM extracts were mixed with POPC vesicles at different ratios, as indicated. The error bars represent the standard error of the mean, n = 3. (For interpretation of the references to colour in this figure legend, the reader is referred to the Web version of this article.)

Using our optimised peroxidase assay protocol, we also tested whether SSBLMs could be formed directly from total (i.e. “crude”) IM extracts overexpressing our protein of interest, since such an approach would prove highly beneficial towards assaying membrane protein targets that are difficult to purify or reconstitute into lipid vesicles. We have previously shown that to deposit solid-supported membranes on planar glass or silica surfaces using crude bacterial membranes, such extracts have to be first mixed with liposomes (e.g. POPC liposomes) to reduce protein content in the membranes [37]. POPC vesicles were thus mixed via the freeze-thaw method with *E*. *coli* IM extracts overexpressing His-tagged NupC at various ratios. Membrane extracts overexpressing the untagged/wild-type construct of NupC were used as negative controls. The results (Fig. 5) confirmed that SSBLMs can also be used as a screening platform when crude IM extracts, containing high protein-to-lipid ratios, are used. The optimum ratio of bacterial IM extracts to POPC liposomes lies between 20 and 40% (w/w), in line with previous findings on planar surfaces [37], suggesting this optimal ratio is independent on the target proteins that is studied. Fig. 5 shows larger values for the standard error of mean when compared to Fig. 4. Comparing individual experiments shows that this is due to varying amounts of target proteins incorporated from the crude membrane extract into the SSBLM, as errors are similar to those in Fig. 4 when ELISAs are performed on the same SSBLM batch. We propose that this is due to the need to mix crude membrane extracts with POPC liposomes, which might result in slight variations in incorporation of membrane proteins into the SSBLMs, even when fixed ratios of POPC versus crude membrane extracts are used.

Although the western blot and ELISA experiments confirmed the presence of His-tagged NupC embedded onto the silica particles, these two methods do not confirm that the lipid bilayers were correctly forming around the particles as compared to, for instance, intact vesicles adsorbing to the surface of the particles. Indeed, the negative effects of membrane proteins on the formation of planar solid supported membranes have previously been documented [38]. We note here, however, that even if some of the vesicles are adsorbed to the spherical silica particles intact, the system would still form a suitable screening platform as indicated by the ELISA results. Nevertheless, in order to further confirm the correct formation of the SSBLMs, as well as to provide a second method to show specific binding to embedded proteins, we also imaged the SSBLM particles via cryo-EM.

**Fig. 6:**
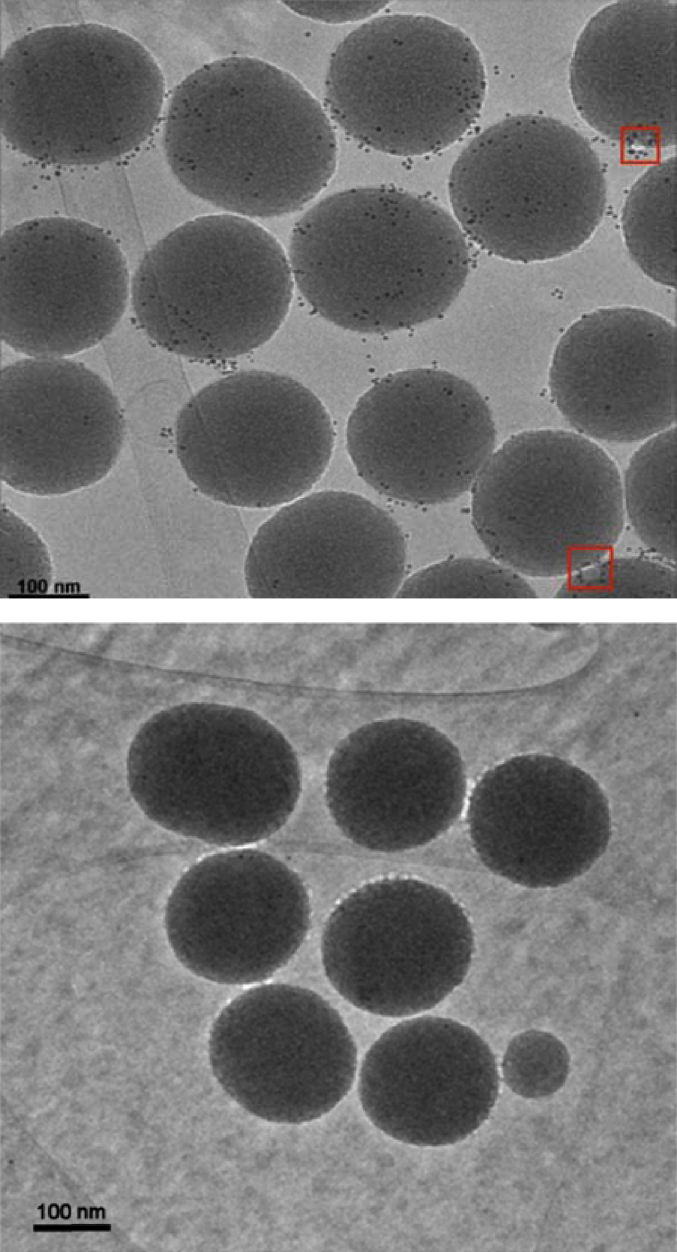
Cryo-EM images of 200 nm silica particles coated with POPC SSBLMs with (top) and without (bottom) embedded His-tagged NupC after incubation with 5 nm gold-conjugated Ni-NTA probes. The two red boxes in the top figure indicate two examples of unfused vesicles. (For interpretation of the references to colour in this figure legend, the reader is referred to the Web version of this article.)

EM samples were prepared using His-tagged NupC/POPC proteoliposomes (and protein-free POPC liposomes for the equivalent negative controls) and subsequently incubated with Ni-NTA-Nanogold probes so as to monitor the distribution of His-tagged NupC (Fig. 6). By rapidly freezing the SSBLMs in vitreous ice, the lipid membrane structure is preserved as opposed to negative staining, which can flatten the specimens being studied. In order to further preserve the quality of the images, a “low-dose” exposure procedure was used such that the electron radiation damage could be minimised. Under these conditions, the discrete lipid membrane components of the SSBLMs could not be directly observed (in contrast to previous studies [15,39]), but the bound Ni-NTA-Nanogold particles clearly indicated the location of the embedded proteins on the surface of the SSBLMs, highlighted by the multitude of representative black “dots”. The images show that Ni-NTA binding was indeed specific to His-tagged NupC within the SSBLMs and that membrane envelope the silica particles to form a SSBLM (i.e., not adsorbed as intact proteoliposomes). We note, however, that for less than 1 in 5 SSBLM particles, unfused vesicles were also visible. Two examples of unfused vesicles are indicated by a red box in Fig. 6.

Analysing a number of EM images, a distribution of 5–60 Ni-NTA-Nanogold were observed per silica particle, with an average of 30 Ni-NTA-Nanogold/silica particle (S.D. = 18). Taking the diameter of the silica particles as 200 nm, the molecular weight of NupC to be 44 kDa, the surface area of POPC as 67 Å^2^ (MW 760 Da), it can be estimated that 130 NupC proteins are present for each silica particle (a 2% (w/w) ratio of NupC to lipid was used to prepare the proteoliposomes). If NupC adopts a random orientation on the silica particles, half of them will have the His-tag facing outwards for the Ni_NTA-Nanogold to bind, i.e. 65 per silica. The lower average number of Ni-NTA-Nanogold that are observed in the EM images could be due to incomplete binding of the Ni-NTA-Nanogold to the exposed his-tags or loss of NupC during the reconstitution into proteoliposomes.

## Discussion

A particular screening method that finds increasing use in both the pharmaceutical and biotechnological fields is that based on phage display. In principle, phage display screening can be performed using detergent-solubilised membrane protein targets. However, detergent-based screening methods come with their own drawbacks, including target denaturation over long periods of storage or the inability to solubilise certain membrane protein classes due to monomer packing defects resulting in their aggregation and, ultimately, inactivation following purification [40]. A final problem with phage display screening against membrane protein targets is the immobilisation strategy. Globular proteins are typically adsorbed onto polymeric or streptavidin coated surfaces. However, detergent solubilisation of membrane proteins and the aforementioned problems with tagging can impede these strategies. Several alternative strategies have been described, such as whole cell panning [41] or embedding the proteins into nanodiscs [42]. Whole cells provide a very complex environment for screening while nanodiscs still require the membrane proteins to be purified to a high yield and purity.

By combining the attractive properties of both submicron materials and model membranes, our proposed SSBLM particles aim to become an improved antigen presentation method available to membrane protein researchers. The successful embedding of NupC within the SSBLM format on both 100 and 200 nm silica particles, along with confirming the accessibility of the embedded proteins towards high-affinity antibody binding, both suggest that the SSBLMs could constitute a promising new means of studying membrane proteins in the future. SSBLMs represent a versatile model system that not only mimics the original cellular lipid environment, but also elegantly circumvents the numerous disadvantages offered by traditional detergent solubilisation methods. Although the ‘shelf-life’ stability of the SSBLM was not studied here, membrane proteins have previously been determined to be stable of weeks in silica-supported membrane systems [43]. Therefore, we believe that the platform could ultimately serve as an enhanced screening support for the discovery of novel antibody binders in an industrial setting, using high-throughput technologies, just as it has already been considered for the role of delivering therapeutic payloads to membrane protein targets via SSBLM-based nanovectors [39].

We note here that suitable liposome/particle ratios must be met in order to avoid partially covered substrates, which can result in non-specific binding. Just as importantly, the blocking of non-specific binding sites and the washing of unbound materials must both be carefully considered in order to reduce the chances of obtaining false positive results. To this end, the format would greatly benefit from a faster washing procedure and one promising alternative would be to assemble the SSBLMs on iron oxide-core, silica-shell particles, so as to facilitate their magnetic separation from solution and thus eliminate the platform's reliance on the more time-consuming centrifugation-based pelleting. Superparamagnetic ferrite particles have already been covered with lipid bilayers in the past [39] and an added benefit of other such improvements would be the possibility for further automation offered by an industrial setting, which would ultimately allow the SSBLMs to be used in high-throughput scenarios as well.

Other improvements could be considered for the use of SSBLMs in screening assays. Some approaches enable the oriented reconstitution of appropriately-tagged membrane proteins (e.g. His-tagged proteolipid bilayers deposited onto Ni-NTA-treated surfaces [44]), while others are better suited at preserving the functionality of the target proteins post-purification (e.g. SSBLMs for electrophysiology [45,46] or electrochemistry [[47], [48], [49], [50], [51], [52], [53]]). Any contact with a solid support can potentially affect protein fluidity across the model membrane and, consequently, prevent the uniform distribution of the designated antigen throughout the chosen screening format. Several alternatives to the conventional method of solid supported membrane formation via proteoliposomal deposition have been trialled in an attempt at bypassing the problems caused by protein immobility or improper membrane solubilisation using detergents [7], such as the self-insertion of purified membrane proteins into an already-formed solid supported membrane [15] or the formation of a polyethylene glycol (PEG)-supported bilayer [[54], [55], [56]].

## Conclusion

In conclusion, we have demonstrated that SSBLMs represent a promising platform for screening assays, where membrane protein targets are displayed embedded within a native-like lipid environment. We have also demonstrated that SSBLMs can be quickly and easily formed using purified proteins reconstituted into liposomes, as well as by directly employing crude membrane extracts. Here, the potential suitability of the SSBLM platform towards high-affinity antibody binding was established using ELISAs and cryo-EM imaging, where the former technique showed that non-specific binding can be minimised through suitable assay modifications. We are now investigating whether the SSBLM can be applied in phage display screening.
